# Prolyl endopeptidase remodels macrophage function as a novel transcriptional coregulator and inhibits fibrosis

**DOI:** 10.1038/s12276-023-01027-8

**Published:** 2023-07-03

**Authors:** Shuang-Zhe Lin, Wei-Jie Wu, Yu-Qing Cheng, Jian-Bin Zhang, Dai-Xi Jiang, Tian-Yi Ren, Wen-Jin Ding, Mingxi Liu, Yuan-Wen Chen, Jian-Gao Fan

**Affiliations:** 1 Department of Gastroenterology, Xin Hua Hospital affiliated to Shanghai Jiao Tong University School of Medicine, Shanghai, 200092 China; 2 Gastrointestinal Endoscopy Center, Fujian Provincial Hospital South Branch, Fuzhou, 350003 Fujian China; 3 State Key Laboratory for Diagnosis and Treatment of Infectious Diseases, National Clinical Research Center for Infectious Diseases, Collaborative Innovation Center for Diagnosis and Treatment of Infectious Diseases, The First Affiliated Hospital, Zhejiang University School of Medicine, Zhejiang University, Hangzhou, 310058 Zhejiang China; 4 Shanghai Key Laboratory of Pediatric Gastroenterology and Nutrition, Shanghai, 200092 China; 5 State Key Laboratory of Reproductive Medicine, The Affiliated Taizhou People’s Hospital of Nanjing Medical University, Taizhou School of Clinical Medicine, Nanjing Medical University, Nanjing, 211166 Jiangsu China; 6 Department of Gastroenterology, Huadong Hospital Affiliated to Fudan University, Shanghai, 200040 China; 7 Department of Gerontology, Huadong Hospital Affiliated to Fudan University, Shanghai, 200040 China

**Keywords:** Transcriptional regulatory elements, Kupffer cells, Liver fibrosis

## Abstract

Macrophages are immune cells crucial for host defense and homeostasis maintenance, and their dysregulation is involved in multiple pathological conditions, such as liver fibrosis. The transcriptional regulation in macrophage is indispensable for fine-tuning of macrophage functions, but the details have not been fully elucidated. Prolyl endopeptidase (PREP) is a dipeptidyl peptidase with both proteolytic and non-proteolytic functions. In this study, we found that *Prep* knockout significantly contributed to transcriptomic alterations in quiescent and M1/M2-polarized bone marrow-derived macrophages (BMDMs), as well as aggravated fibrosis in an experimental nonalcoholic steatohepatitis (NASH) model. Mechanistically, PREP predominantly localized to the macrophage nuclei and functioned as a transcriptional coregulator. Using CUT&Tag and co-immunoprecipitation, we found that PREP was mainly distributed in active *cis*-regulatory genomic regions and physically interacted with the transcription factor PU.1. Among PREP-regulated downstream genes, genes encoding profibrotic cathepsin B and D were overexpressed in BMDMs and fibrotic liver tissue. Our results indicate that PREP in macrophages functions as a transcriptional coregulator that finely tunes macrophage functions, and plays a protective role against liver fibrosis pathogenesis.

## Liver fibrosis: Protective role of enzyme uncovered

An enzyme that exerts a protective effect against the development of liver fibrosis could provide a promising therapeutic target. Macrophages are innate immune cells that are also critical for maintaining the balance of tissue development and repair. Dysregulation in macrophages can trigger multiple conditions, including liver fibrosis, the excessive thickening and scarring of the organ’s tissues. Shuang-Zhe Lin at Shanghai Jiao Tong University School of Medicine, China, and co-workers demonstrated that the prolyl endopeptidase enzyme (PREP) localises to the nuclei of macrophages and regulates macrophage functions. PREP binds directly to a protein regulating gene expression, thereby influencing the production of profibrotic proteins, and protects against the development of fibrosis. Knocking out the *Prep* gene in a mouse model of nonalcoholic fatty liver disease led to altered macrophage functioning and aggravated liver fibrosis.

## Introduction

Macrophages are essential phagocytic immune cells that extensively exist in various mammalian solid organs, such as the liver, lung and brain^1^. They not only play a vital role in host innate immune defense against infection, but also irreplaceably participate in tissue development, homeostasis maintenance and tissue repair^2,3^. Correspondingly, dysregulations in macrophage function can promote pathological processes such as uncontrolled inflammation and aberrant extracellular matrix (ECM) remodeling, and have been implicated in the pathogenesis of liver, lung and brain diseases^4–6^. The cellular state and functions of macrophages are largely subjected to epigenetic regulation of gene expression. Extensive epigenetic reprogramming of macrophage *cis*-regulatory information and transcription factor binding have been identified in M1/M2 macrophage polarization and tissue microenvironment-induced macrophage differentiation^7–9^. Targeting epigenetic molecules in macrophages may become a promising therapeutic approach to diseases such as liver fibrosis, atherosclerosis and neurologic disorders^10–12^.

According to current theories, key transcription factors in macrophages have been preliminarily identified and roughly categorized into two types, lineage-determining transcription factors (LDTFs, e.g., PU.1 and C/EBPs) and signal-dependent transcription factors (SDTFs, e.g., NF-κB and IRFs), which function in the basal activation of downstream genes and further activation of downstream genes in response to external stimuli, respectively^9,13,14^. However, the details of molecular mechanisms underlying epigenetic regulation have not been fully elucidated. RNA polymerase physically interacts with transcription factors that directly bind to DNA, as well as transcriptional coregulators that do not directly bind DNA, forming nuclear condensates that play regulatory roles in transcription initiation and elongation^15,16^. The composition of these nuclear condensates has been only partially reported, and there should be other undiscovered proteins with important transcription regulatory activity in the nuclear condensates^17^.

Prolyl endopeptidase (PREP) is a member of the dipeptidyl peptidase (DPP) family with the capacity to cleave small peptides (e.g., peptide-like hormones and neuropeptides) at the carboxyl side of an internal proline residue^18,19^. It can also physically interact with various proteins to mediate nonproteolytic functions, such as facilitating α-synuclein formation in Parkinson’s disease (PD) and regulating synaptic plasticity in neurons^20,21^. PREP is highly expressed in the brain, testis and liver^22,23^ and has also been detected in myeloid cells, including macrophages^23,24^. In our previous studies, hepatic inflammation was alleviated after *Prep* ablation in murine nonalcoholic steatohepatitis (NASH) models, as manifested by decreased infiltration of macrophages and neutrophils^25,26^. Considering the central role of liver macrophages in orchestrating liver inflammation^5,27^, we postulate that PREP might influence macrophage states and functions.

In this study, we found that PREP exerts a transcription-based regulatory effect on macrophage function. PREP in macrophages predominantly localizes to the nucleus where it modulates the transcriptome in quiescent and M1/M2-polarized macrophages. Furthermore, we found a protecting role of PREP against fibrosis in an experimental NASH model. Mechanistically, we demonstrated that PREP functions as a transcriptional coregulator via physical interaction with the transcription factor PU.1 and directly regulates a large set of active *cis*-regulatory genomic regions. Among the regulated downstream genes, the expression of genes encoding profibrotic cathepsin B and D was suppressed by PREP in macrophages, explaining the protective role of PREP against liver fibrosis. Altogether, our study is the first report indicating a noncanonical molecular function of PREP as a transcriptional coregulator in macrophages. Our data highlight the profound physiological value of PREP-mediated transcriptional regulation in reprogramming macrophage functions and help to deepen current understanding of fibrosis pathogenesis.

## Materials and Methods

### Animal experiments

*Prep*^-/-^ mice and their heterozygous littermates (*Prep*^+/-^ mice) with a C57BL6/J background were generated as previously described^25^. A NASH-related fibrosis model (WD/CCl_4_) was established according to previously described research^28^ with modifications: 12-week-old mice were fed a western diet (a high-fat and high-cholesterol diet comprising 88% standard diet, 10% lard and 2% cholesterol by weight, along with a high-sugar solution containing 23.1 g/L fructose and 18.9 g/L glucose), and were intraperitoneally injected with a carbon tetrachloride (CCl_4_) and corn oil (1:19) mixture at a dose of 4 μl/body weight (g) once per week. The control group mice (ND/Oil) were fed a normal chow diet and normal tap water, and were intraperitoneally injected with corn oil (the control vehicle) once per week. The mice were housed in a 12-hour light/dark cycle in a temperature-controlled room under SPF conditions, and maintained for 12 weeks with free access to food and water. At the end of the experimental period, the mice were weighed and fasted for 12 hours with free access to water before measuring fasting blood glucose. The mice were euthanized at 12 weeks by exsanguination after CO_2_ anesthesia, and liver and serum samples were then collected.

### Cell culture and polarization

To derive bone marrow-derived macrophages (BMDMs), bone marrow cells freshly isolated from the femora of *Prep*^-/-^ and *Prep*^+/-^ littermates were seeded in 6-well plates, and cultivated in RPMI 1640 supplemented with 10% FBS (Gibco, 16000-044), a penicillin/streptomycin mix (Gibco, 15140122) and 50 ng/ml M-CSF (PeproTech, AF-315-02) at 37 °C with 5% CO_2_. BMDMs were fully differentiated and ready for use on Day 7. For macrophage polarization experiments, BMDMs were left unstimulated, stimulated with 50 ng/ml LPS, or stimulated with 20 ng/ml murine Interleukin-4 (PeproTech, AF-214-14) and 20 ng/ml murine Interleukin-13 (PeproTech, AF-315-02) for 24 h. RAW 264.7 cells were obtained from the China Center for Type Culture Collection (Shanghai, China) and cultured and maintained in DMEM supplemented with 10% FBS at 37 °C with 5% CO2. NIH-3T3 cells were purchased from Procell Life Science Technology Co., Ltd. (Wuhan, China) and were cultured and maintained in DMEM supplemented with 10% CS (AusGeneX, NCS-S) at 37 °C with 5% CO2.

### RNA-seq data analysis pipeline

Raw reads in fastq format of RNA-seq were first processed using Trimmomatic (v0.36), and low-quality reads were removed to obtain clean reads for subsequent analyses. The clean reads were aligned to the mouse genome (GRCm39) using hisat2 (v2.2.1.0) with default parameter settings. The aligned reads were assembled into transcripts using stringtie2 (v1.3.3b). The resulting count tables were passed to R (v4.1.2) for further analysis.

Consistency between biological replicates was checked using principal component analysis (PCA) by using the function prcomp from the R package stats (v4.1.2), and visualized by the gg3D package (v0.0.0.9000). Differentially expressed genes (DEGs) were assessed with DESeq2 (v1.34.0) based on the criteria of p-adj (adjusted *p* value) < 0.05 and FC (fold change) > 1.5. Volcano plots made by the package ggplot2 (3.3.6) and hierarchically clustered heatmaps made by package pheatmap (v1.0.12) were used to visualize DEGs. A gene set enrichment analysis (GSEA) of the KEGG pathways based on DESeq2 outputs was performed and visualized using the package clusterProfiler (v4.2.2).

### CUT&Tag library preparation and sequencing

NovoNGS® CUT&Tag 3.0 High-Sensitivity Kit (Novoprotein, Shanghai, China, N259) was used for CUT&Tag library preparation according to the manufacturer’s recommendations. Briefly, 1 × 10^5^ cells were bound to ConA–coated magnetic beads and sequentially incubated with a primary rabbit anti-prolyl endopeptidase (1:100, Abcam, ab58988) and ChiTag goat anti-rabbit IgG antibody (1:200). The cells were then incubated with pAG-Tn5, followed by tagmentation at 37 °C and heat inactivation at 55 °C. DNA fragments were extracted and amplified. After DNA purification with DNA Clean Beads, libraries were sequenced on an Illumina NovaSeq 6000 for the generation of 150-bp paired-end reads.

### CUT&Tag-seq data analysis pipeline

Paired-end reads of CUT&Tag-seq were aligned to mm10 genome using Bowtie2 (v2.3.4.3) with options: -end-to-end -sensitive. DeepTools (3.3.2) was used to generate the read coverage track (command bamCoverage with normalization by RPKM), calculate scores per genome region (command computeMatrix) and perform subsequent visualization (command plotProfile). The RPKM-normalized CUT&Tag read coverage tracks were visualized via IGV browser (v2.9.4) tracks.

For peak calling of CUT&Tag-seq data, bigWig files containing RPKM-normalized CUT&Tag signal produced by bamCoverage were first converted to bedGraph files by bigWigToBedGraph in the UCSC Toolkit. Peak calling was then performed using the command bdgpeakcall in macs2 (v2.1.4) with the cutoff options (-c 160 -l 320 -g 20). Peak annotations and metagene profiles were performed with the R package ChIPseeker (v1.30.3). De novo motif enrichment analysis of PREP CUT&Tag peaks was performed using the findMotifsGenome.pl program in the HOMER software suite^29^ with default options. Motif visualization was performed by using the R package ggseqlogo (v0.1).

### BETA analysis

Binding and expression target analysis (BETA, v1.0.7)^30^ was performed to predict the potential activating or repressive function of PREP and potential downstream targets by combining the CUT&Tag–seq and RNA-seq results. Peak interval files obtained with macs2 and differential analysis results obtained with DESeq2 were used as inputs with the filtering options (-df 0.01 -d 25000).

### Statistical analysis

Data are presented as mean ± standard error (SEM). Statistical analysis was performed using R (v4.1.2). Differences between two groups were analyzed by unpaired two-tailed Student’s t test, and one-way ANOVA and LSD (least significant difference) post hoc test were used to compare differences among multiple groups. *P* values are denoted by *P < 0.05 and **P < 0.01. Exact *P* values were calculated by R unless specified differently in the figure legend.

## Results

### PREP profoundly altered the transcriptomic landscape and functional state of both quiescent and activated macrophages

**Fig. 1:**
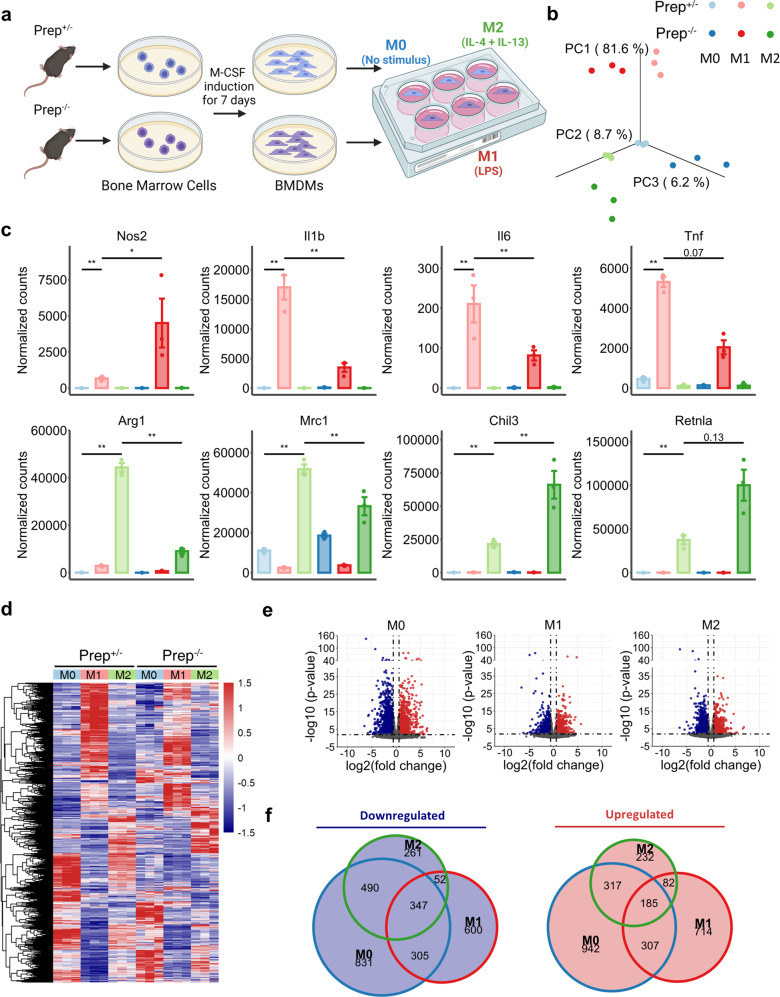
*Prep* gene ablation significantly alters the transcriptome in unpolarized (M0) and M1/M2-polarized murine bone marrow-derived macrophages (BMDMs). **a** Strategy used to generate differentially polarized BMDMs from *Prep*^+/-^ and *Prep*^-/-^ mice (created with BioRender.com). **b-e** Bulk RNA-seq data obtained from differentially polarized BMDMs of *Prep*^+/-^ and *Prep*^-/-^ mice. A Principal component analysis (PCA) was performed (**b**). Bar plots showing representative gene expression of M1/M2 polarization markers, with significance symbols representing the p-adj value obtained from DESeq2 (**c**). Differentially expressed genes (DEGs) between *Prep*^-/-^ and *Prep*^+/-^ BMDMs (*Prep*^-/-^ vs. *Prep*^+/-^) in the M0, M1 or M2 state are presented in a heatmap (**d**) and volcano plots (**e)**. Each group comprised three biological replicates. **f** Venn diagram showing the overlapping *Prep* knockout-induced upregulated or downregulated DEGs among M0, M1 and M2 BMDMs. Data information: Bars represent the mean ± SEM. *p < 0.05, **p < 0.01.

To examine whether PREP participates in the functional regulation of macrophages, we obtained BMDMs from *Prep*^-/-^ mice and heterozygous littermates, and performed a parallel comparative analysis of global mRNA expression in unstimulated quiescent (M0), LPS-stimulated (M1), and IL-4&IL-13-stimulated (M2) BMDMs by bulk RNA-seq (Fig. 1a). Both LPS stimulation and IL-4&IL-13 stimulation induced large transcriptomic changes in the *Prep*^+/-^ and *Prep*^-/-^ BMDMs (Fig. 1b, Supplementary Fig. 1a), including significantly altered expression of M1 (*Nos2*, *Il1b*, *Il6* and *Tnf*) and M2 (*Arg1*, *Mrc1*, *Chil3* and *Retnla*) markers (Fig. 1c), indicating the successful induction of M1/M2 polarization in BMDMs. More importantly, we found that the transcriptomes of M0, M1 and M2 BMDMs were all significantly changed by *Prep* knockout as manifested by numerous DEGs (Fig. 1c–e). For the arginine metabolism-related M1/M2 marker pair, *Prep* ablation promoted the upregulation of *Nos2* and the downregulation of *Arg1* during M1 and M2 polarization, respectively. The phagocytosis-related M2 marker *Mrc1* was also downregulated after *Prep* ablation. In contrast, the expression of the classical inflammatory triad (*Il1b*, *Il6* and *Tnf*) during M1 polarization as downregulated after *Prep* ablation, while the expression of the M2 markers *Chil3* and *Retnla* was upregulated, indicating a heterogeneous effect of PREP in modulating the expression of M1 and M2 markers during macrophage polarization.

We further examined the relationship between these DEG subsets in macrophages in different states. The gene sets of *Prep* knockout-induced upregulated DEGs in the M0/M1/M2 BMDMs included a considerable number of shared genes (82 ~ 307 genes shared by two cell states and 185 by all three cell states), while 232 ~ 942 genes were exclusively in gene set of one cell state (Fig. 1f). Similarly, among the *Prep* knockout-induced downregulated gene sets in M0/M1/M2 BMDMs, a considerable number of genes were shared (52 ~ 490 genes were shared by two cell states and 347 by all three cell states), while 261 ~ 831 genes were exclusively in gene set of one cell state (Fig. 1f). In contrast, only a few of the *Prep* knockout-induced upregulated genes found in one cell state (i.e., M0/M1/M2) were found to be downregulated by *Prep* knockout in the other two cell states, and vice versa (Supplementary Fig. 1b).

### PREP inhibits liver fibrosis in an experimental NASH model

**Fig. 2:**
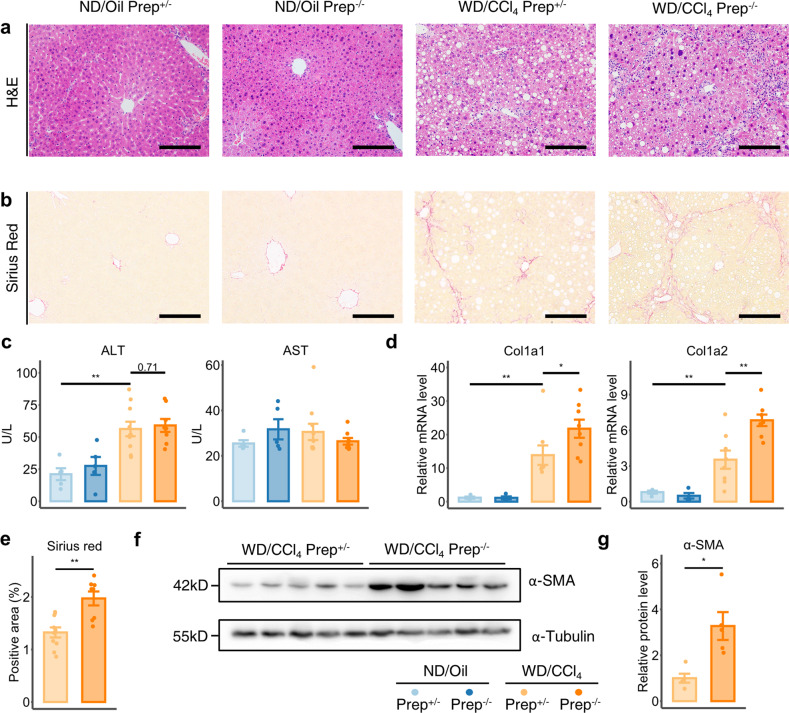
*Prep* gene ablation exacerbates WD/CCl_4_-induced murine liver fibrosis. **a, b** Representative image of H&E-stained (**a**) and Sirius red-stained (**b**) liver sections of *Prep*^+/-^ and *Prep*^-/-^ mice treated with WD/CCl_4_ or ND/Oil. Scale bar indicates 100 μm. **c** Serum ALT and AST levels of *Prep*^+/-^ and *Prep*^-/-^ mice treated with WD/CCl_4_ or ND/Oil. The results were compared via one-way ANOVA and LSD post hoc test. *n* = 10 for WD/CCl4-treated *Prep*^+/-^ mice group; *n* = 8 for WD/CCl_4_-treated *Prep*^-/-^ mice group; *n* = 5 for ND/Oil-treated groups. (**d**) Relative mRNA levels of *Col1a1* and *Col1a2* in *Prep*^+/-^ and *Prep*^-/-^ mice treated with WD/CCl_4_ or ND/Oil. The results were compared by one-way ANOVA and LSD post hoc test. **e** Semiquantitative analysis of Sirius red-stained areas in *Prep*^+/-^ and *Prep*^-/-^ mice treated with WD/CCl_4_. The results were compared by unpaired two-tailed Student’s t test. *n* = 10 for *Prep*^+/-^ mice group; *n* = 8 for *Prep*^-/-^ mice group. **f, g** Western blot analysis (**f**) was performed on liver tissue lysates of *Prep*^+/-^ and *Prep*^-/-^ mice treated with WD/CCl_4_ for α-smooth muscle actin (α-SMA) and the housekeeping control α-tubulin; densitometry results (**g**) were normalized to the level of α-tubulin and compared by unpaired two-tailed Student’s t test. *n* = 5 for each group. Data information: Bars represent the mean ± SEM. *p < 0.05, **p < 0.01.

To determine whether PREP-mediated alterations in macrophage function were pathophysiologically significant under fibrotic conditions, we established a murine NASH model with rapidly progressing and extensive fibrosis^28^. After 12 weeks of feeding, the key histological features of NASH, including hepatic steatosis, inflammation (Fig. 2a), and extensive fibrosis (Fig. 2b), were recapitulated in this mouse model. Elevation in serum ALT was found (Fig. 2c) in this model, although the level of serum AST was not significantly changed. Mouse body weight was decreased, possibly due to the toxicity of CCl_4_ (Supplementary Fig. 2a-b), while other metabolic indices, including fasting blood glucose level, liver weight and epididymal fat weight, were not significantly altered (Supplementary Fig. 2b).

We then compared *Prep*^-/-^ mice with *Prep*^+/-^ mice under WD/CCl_4_ treatment and found that liver fibrosis, as assessed by collagen gene expression, Sirius red staining, and α-smooth muscle actin (α-SMA) protein level, was significantly increased in the *Prep*^-/-^ mice (Fig. 1d–g), indicating increased liver fibrosis after *Prep* knockout. In contrast, no significant change in serum ALT level (Fig. 1c) was found in the WD/CCl_4_-treated *Prep*^-/-^ mice, indicating that *Prep* knockout did not exacerbate hepatocellular injury in the WD/CCl_4_ model mice.

### PREP is predominantly localized to the nucleus in macrophages and is dynamically distributed in close proximity to active *cis*-regulatory DNA sequences

**Fig. 3:**
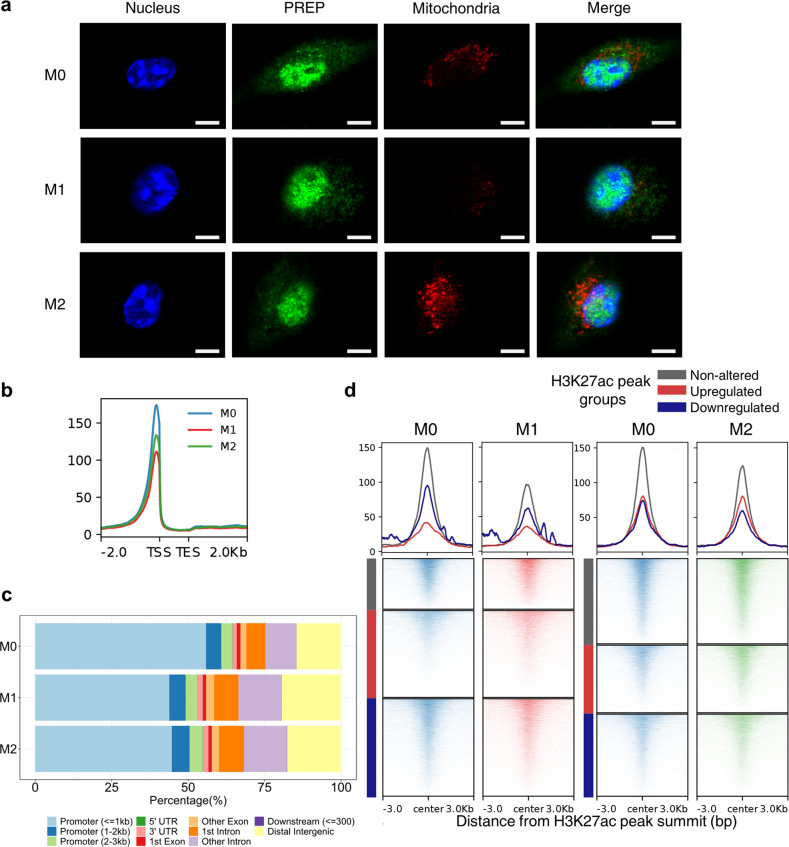
Prolyl endopeptidase is predominantly localized in the nuclei of macrophages and shows dynamic chromatin distribution during M1/M2 polarization. **a** Confocal immunofluorescence images showing the subcellular localization of prolyl endopeptidase (PREP) in BMDMs in the M0, M1 or M2 state. Nuclei and mitochondria were labeled with DAPI and MitoTracker Red, respectively. Scale bar indicates 5 μm. **b** The average enrichment profile of PREP CUT&Tag signals ± 2 kb around genic regions of BMDMs. TSS, transcription start site; TES, transcription end site. **c** Barplot showing the PREP CUT&Tag peak distribution among different genomic features. **d** The average enrichment profile and heatmap showing PREP CUT&Tag signals ± 3 kb around H3K27ac ChIP peaks classified by alterations during M1/M2 polarization of BMDMs.

To clarify the mechanism underlying the marked PREP-mediated transcriptomic changes in macrophages, we performed immunofluorescence assay to explore the subcellular localization of PREP in M0/M1/M2 BMDMs. Mitochondria were also labeled to examine the potential localization of PREP to mitochondria, which had been indicated in a previous report^31^. Unexpectedly, we found that PREP in BMDMs mainly localized in nuclei, regardless of the functional state of macrophage, and did not colocalize with heterochromatin (dense DAPI-stained areas); some immunofluorescence signals of PREP were found in the cytoplasm, where they formed a dotted distribution pattern, but did not colocalize with mitochondria (Fig. 3a). Similar results were also found in the RAW264.7 murine macrophage cell line (Supplementary Fig. 3a).

To explore the potential distribution of nuclear PREP throughout the genomic DNA, we performed CUT&Tag-seq of PREP in M0/M1/M2 BMDMs. We found that PREP CUT&Tag-seq signals (Fig. 3b) and called peaks (Supplementary Fig. 3b) were enriched in genomic areas around transcription start sites (TSSs). The distribution intensity of PREP signals in TSS-adjacent regions were weaker after M1 or M2 polarization (Fig. 3b). Genomic feature annotation also revealed that most PREP CUT&Tag peaks were enriched at promoters (Fig. 3c). These results indicated that PREP distribution in the nucleus was closely associated with *cis*-regulatory sequences, as exemplified by promoters.

Acetylation of histone H3 lysine 27 (H3K27ac) marks promoters and enhancers in the activated state, and the enrichment of this mark is positively correlated with the transcriptional regulatory activity of its deposition site^32,33^. To further examine whether PREP distribution in the genome is related to active promoters and enhancers, we obtained ChIP-seq data of H3K27ac in BMDMs from a previous study^7^, and classified the H3K27ac peaks into non-altered, upregulated and downregulated peaks according to changes in H3K27ac deposition during M1 or M2 polarization (Supplementary Fig. 3c). We found that PREP was distributed in genomic regions centered by H3K27ac peaks, and most of these regions represented non-altered H3K27ac peaks during either M1 or M2 polarization (Fig. 3d), indicating preferentiality of PREP distribution to active *cis*-regulatory DNA sequences that showed no alteration in transcriptional regulatory activity. In addition, PREP distribution on genomic regions centered by nonaltered and downregulated H3K27ac peaks after either M1 or M2 polarization was relatively diminished (Fig. 3d).

### PREP is a transcriptional coregulator that interacts with PU.1

**Fig. 4:**
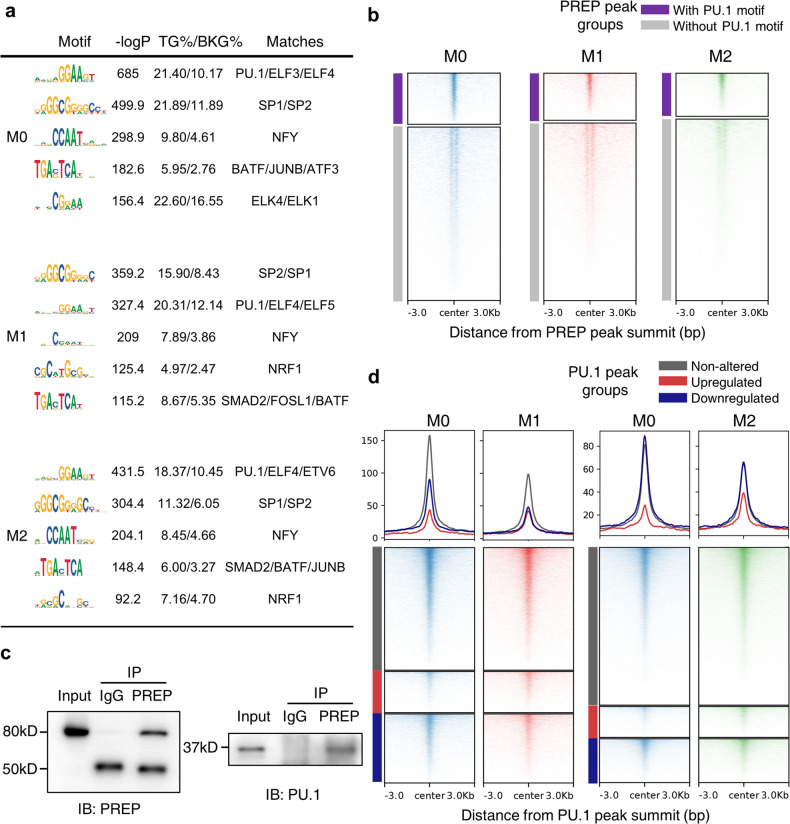
PREP interacts with PU.1. **a** De novo motif enrichment analysis of PREP CUT&Tag peaks using a GC-matched genomic background. The top 5 motifs under each state are shown with summary statistics and the predicted matching transcription factors. **b** The average enrichment heatmap showing PU.1 ChIP signals ± 3 kb around PREP CUT&Tag peaks with/without the PU.1 motif in M0/M1/M2 BMDMs. **c** Coimmunoprecipitation of PREP and PU.1 in RAW264.7 cell protein extracts. **d** The average enrichment profile and heatmap of PREP CUT&Tag signals ± 3 kb around PU.1 ChIP peaks classified by alterations during the M1/M2 polarization of BMDMs.

To characterize the genomic distribution pattern of PREP and identify potentially related transcription factors, we performed a de novo DNA motif analysis of PREP peak regions. We found that the enriched motifs across different PREP CUT&Tag peak sets were similar, especially between M0 and M2 (Fig. 4a). The predicted matching transcription factors related to the top de novo motifs included PU.1/ELFs, SPs, NFY, etc. (Fig. 4a), indicating possible molecular interactions between PREP and transcription factors during transcriptional regulation. To verify the interaction between PREP and PU.1 (the master LDTF in macrophages), we first classified the PREP CUT&Tag peaks into two sets according to whether the peak sequence contained the PU.1 motif found in de novo DNA motif analysis, and explored the relationship between the PU.1 ChIP-seq signal distribution (published in a previous study^7^) and different sets of PREP CUT&Tag peaks. We found that PREP peaks with the PU.1 motif were enriched with PU.1 distribution, while PREP peaks without PU.1 motif exhibited scarce distribution of PU.1 (Fig. 4b). Then, we performed a coimmunoprecipitation assay with RAW264.7 cells and found that PU.1 physically interacted with PREP in macrophages (Fig. 4c). We also explored the distribution relationship between PREP CUT&Tag signals and different sets of PU.1 peaks (Supplementary Fig. 3d), and found that PREP was distributed in extremely close proximity to PU.1 peaks, particularly non-altered PU.1 peaks during M1/M2 polarization (Fig. 4d). These results collectively indicate that the interaction between PU.1 and PREP is involved in PREP-mediated transcriptional regulation.

### PREP-mediated transcriptional regulation directly remodels the transcriptome and functions of macrophages

**Fig. 5:**
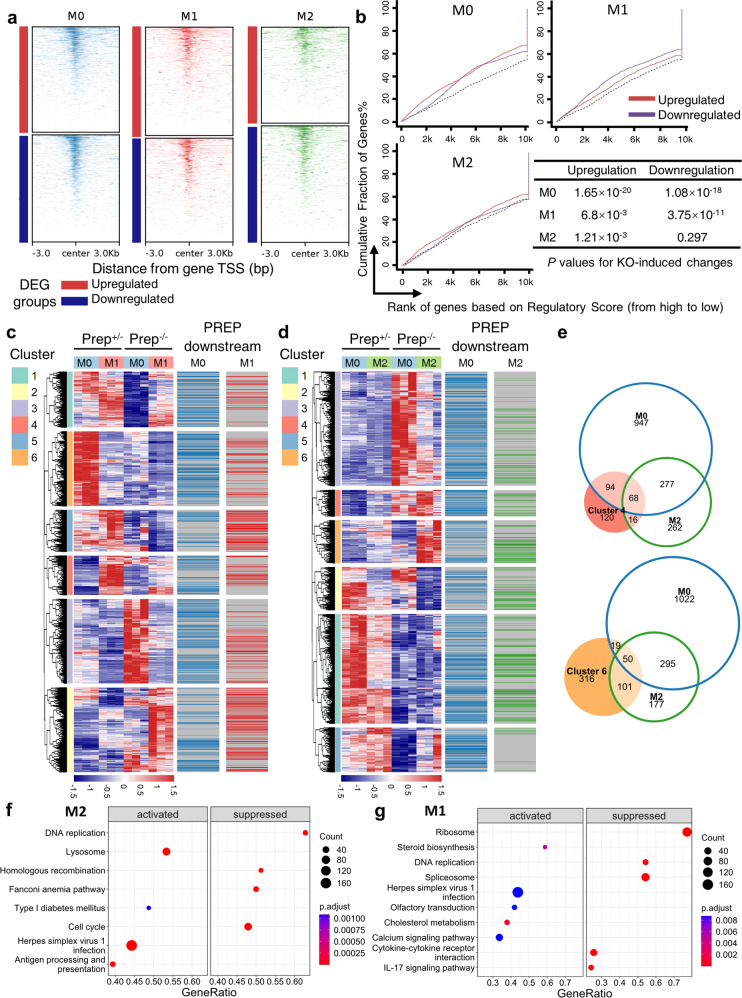
The influence of PREP on the macrophage transcriptome is associated with PREP-mediated direct transcriptional regulation. **a** Heatmap showing the average enrichment of PREP CUT&Tag signals ± 3 kb around the TSS of upregulated and downregulated DEGs in the M0/M1/M2 state. **b** Activating/repressive function prediction of the PREP CUT&Tag peaks in M0/M1/M2 BMDMs by BETA (binding and expression target analysis) using PREP CUT&Tag-seq (in *Prep*^+/-^ BMDMs in the M0/M1/M2 state) and RNA-seq (of *Prep*^-/-^ and *Prep*^+/-^ BMDMs in the M0/M1/M2 state). The genes are cumulated by rank on the basis of the regulatory potential score from high to low, according to PREP CUT&Tag-seq data. The cumulative fractions of upregulated, downregulated and unaffected (used as the background) genes after *Prep* knockout as determined by RNA-seq are denoted by the red, purple and black lines, respectively. *P* values for activating/repressive function prediction were determined by Kolmogorov–Smirnov test. **c, d** Heatmap illustrating the differentially expressed genes (DEGs) between *Prep*^-/-^ and *Prep*^+/-^ BMDMs (*Prep*^-/-^ vs. *Prep*^+/-^) in M0 and M2 states (**c**), as well as in M0 and M1 states (**d**). The hierarchical clustering based on RNA-seq data, together with directly PREP-regulated downstream genes identified by BETA, is annotated on the left side of the heatmap. (**e**) Venn diagrams showing overlapping genes between clusters 4 and 6 according to hierarchical clustering and PREP-regulated direct downstream genes in BMDMs in the M0 and/or M2 state. **f**, **g** Dotplot summarizing the results from gene set enrichment analysis (GSEA) of KEGG pathways enriched in DEGs between *Prep*^-/-^ and *Prep*^+/-^ BMDMs (*Prep*^-/-^ vs. *Prep*^+/-^) in the M2 state (**f**) and M1 state (**g**).

To clarify the overall impact and corresponding downstream genes of PREP-mediated transcriptional regulation, we first explored the distribution relationship between PREP CUT&Tag signals ± 3 kb around the TSS of upregulated and downregulated DEGs in the M0/M1/M2 BMDMs, and found a substantial distribution of PREP in close proximity to the TSS of some DEGs (Fig. 5a). For further identification of the direct downstream genes of PREP out of other indirectly affected downstream DEGs, we incorporated PREP CUT&Tag-seq data and RNA-seq data via binding and expression target analysis (BETA)^30^. PREP exhibited an overall suppressive effect (*Prep* knockout-induced upregulation) in M0, M1 and M2 BMDMs, as well as an overall activating effect (*Prep* knockout-induced downregulation) on transcription in M0 and M1 BMDMs (Fig. 5b). We also explored the relationship between PREP downstream genes identified by BETA and gene clusters identified by hierarchical clustering of DEGs, and found that PREP downstream genes were differentially enriched among *Prep* knockout-induced DEG clusters with different expression patterns in M0/M1/M2 BMDMs (Fig. 5c, d). These results collectively indicate that PREP exerts direct transcriptional regulation on different gene sets in both quiescent (M0) and M1/M2-polarized macrophages.

Macrophages in a fibrotic microenvironment often exhibit M2-like molecular characteristics^34^. To further explore the underlying mechanism of *Prep* knockout-induced exacerbated fibrosis, we focused on gene clusters 4 and 6 obtained via transcriptome analysis of M0 and M2 cells, which were characterized by upregulated expression after *Prep* knockout and/or M2 polarization (Fig. 5d). We found that PREP direct downstream genes in M0 and/or M2 BMDMs constituted a substantial proportion of these two clusters (Fig. 5e), indicating a prominent role of PREP in direct inhibition of gene expression in M2-polarized macrophages.

We then performed functional enrichment of *Prep* knockout-induced DEGs in M2-polarized BMDMs by performing a GSEA of KEGG pathways, and found that activated pathways induced by *Prep* knockout included “lysosome”, “herpes simplex virus 1 infection” and “antigen processing and presentation”, while suppressed pathways were mainly related to “DNA replication” and related pathways such as “homologous recombination” and “cell cycle” (Fig. 5f). We also performed GSEA of KEGG pathways in *Prep* knockout-induced DEGs M1-polarized BMDMs, and found that the main activated pathways included “steroid biosynthesis” and “cholesterol metabolism”, while the suppressed pathways were mainly related to the central dogma of genetics (i.e., “ribosome”, “DNA replication” and “spliceosome”) and proinflammatory immune activation (“cytokine-cytokine receptor interaction” and “IL-17 signaling pathway”) (Fig. 5g).

### PREP inhibits lysosome-related functions and lysosomal cathepsin expression in macrophages

**Fig. 6:**
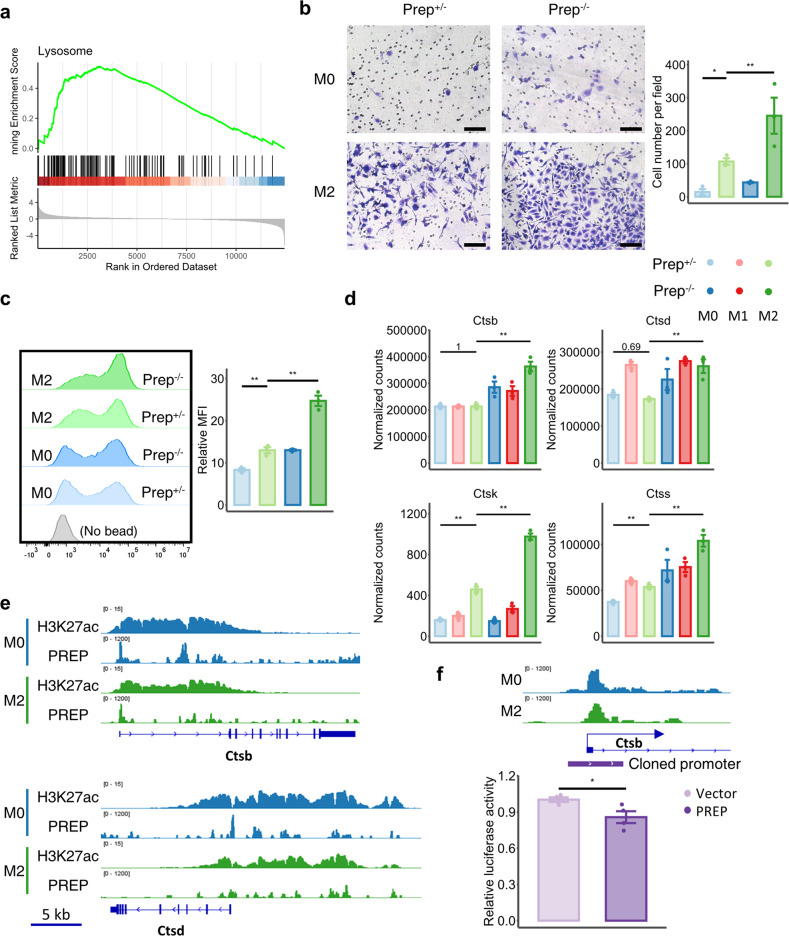
PREP-mediated transcriptional regulation inhibits the expression of cathepsins in M2 -polarized macrophages. **a** Gene set enrichment analysis (GSEA) plot showing the KEGG pathway “lysosome” in the DEGs between the *Prep*^-/-^ and *Prep*^+/-^ BMDMs (*Prep*^-/-^ vs. *Prep*^+/-^) in the M2 state. **b** Phagocytosis assay of the *Prep*^-/-^ and *Prep*^+/-^ BMDMs in the M0/M2 state. The left panel shows representative flow cytometry histograms of FITC fluorescence intensity, and the right panel shows the relative median fluorescence intensity (MFI) in the FITC channel. The results were compared by one-way ANOVA and LSD post hoc test. *n* = 3 for each group. **c** Invasion assay of the *Prep*^-/-^ and *Prep*^+/-^ BMDMs in the M0/M2 state. Scale bar indicates 100 μm. The left panel shows representative microscopic fields, and the right panel shows the cell numbers per field. The results were compared by one-way ANOVA and LSD post hoc test. *n* = 3 for each group. **d** Representative gene expression of cathepsins in the bulk RNA-seq data obtained from differentially polarized BMDMs of *Prep*^+/-^ and *Prep*^-/-^ mice. **e** Representative PREP CUT&Tag peak tracks showing H3K27ac deposition and PREP distribution at the *Ctsb* and *Ctsd*, *Ctsk* and *Ctss* gene loci in BMDMs in the M0 and M2 states. **f** Relative luciferase activity of the *Ctsb* promoter in NIH-3T3 cells transfected with the PREP overexpression plasmid or control plasmid. The results were compared by unpaired two-tailed Student’s t test. *n* = 4 for each group. The relationship between the cloned *Ctsb* promoter and PREP distribution in BMDMs in the M0 and M2 states is shown. Data information: Bars represent the mean ± SEM. *p < 0.05, **p < 0.01.

The top first activated pathway induced by *Prep* knockout in M2 BMDMs was “lysosome”, as indicated by GSEA of KEGG pathways (Figs. 5e and 6a). Considering the central physiological role of lysosomes in phagocytosis and cell invasion^35^, we assessed the phagocytotic and invasion abilities of *Prep*^-/-^ and *Prep*^+/-^ BMDMs in the M0 and M2 states. We found that M2 polarization significantly increased the number of latex beads phagocytosed by BMDMs and the number of invading BMDMs, while *Prep* knockout significantly induced a further increase in phagocytosis and cell invasion in the M2 state (Fig. 6b, c), indicating an inhibitory role of PREP in lysosome-related macrophage phagocytosis and invasion functions. We further evaluated lysosome-related DEGs induced by *Prep* knockout, and found that a substantial portion of genes encoding lysosome catabolic enzymes. These genes include genes encoding cathepsin proteases, i.e., *Ctsb*, *Ctsd*, *Ctsk*, and *Ctss* (Fig. 6d). Only the expression of *Ctsk* and *Ctss* was significantly induced after M2 polarization of *Prep*^+/-^ BMDMs, while the expression of *Ctsb* and *Ctsd* remained unchanged. However, when *Prep* was knocked out, the expression of *Ctsb*, *Ctsd*, *Ctsk* and *Ctss* in M2 BMDMs was consistently increased (Fig. 6d).

To validate the direct transcriptional repression of PREP on the expression of genes encoding cathepsin, we first inspected PREP CUT&Tag signal in BMDMs. We found that the PREP distribution sites were distributed around or inside the cathepsin gene regions, and in close proximity to H3K27ac-marked regions (Fig. 6e). Then, we sought to validate PREP-mediated direct transcriptional repression of *Ctsb* and *Ctsd* in NIH-3T3 cells, a murine cell line used for transfection and previously reported to display nucleus-localized PREP^36^. We found that *Prep* silencing and overexpression in NIH-3T3 cells induced the upregulation and downregulation of Ctsb/Ctsd expression, respectively (Supplementary Fig. 4a-b). Furthermore, we performed a dual-luciferase reporter assay with NIH-3T3 cells, and found that the transcriptional activity of the *Ctsb* promoter was suppressed when PREP was overexpressed (Fig. 6f). These results collectively indicate that PREP-mediated transcriptional regulation inhibits the expression of genes encoding cathepsins.

### The absence of PREP contributes to an increase in profibrogenic cathepsin B and D levels in the fibrotic liver

**Fig. 7:**
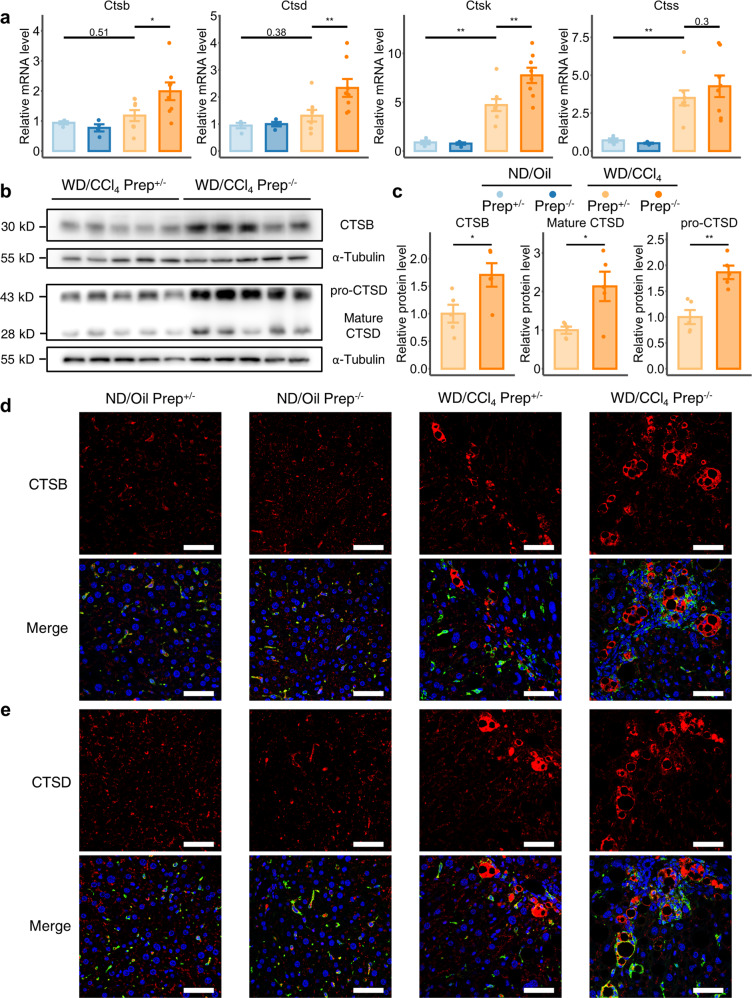
Profibrogenic cathepsin B and D are further upregulated in WD/CCl_4_-induced liver fibrosis after mouse *Prep* gene ablation. **a** Relative mRNA levels of the cathepsin family in *Prep*^+/-^ and *Prep*^-/-^ mice treated with WD/CCl_4_ or ND/Oil. The results were compared by one-way ANOVA and LSD post hoc test. *n* = 8 for WD/CCl_4_-treated groups; *n* = 4 for ND/Oil-treated groups. **b**, **c** Western blot analysis (**b**) performed on liver tissue lysates of *Prep*^+/-^ and *Prep*^-/-^ mice treated with WD/CCl_4_. The level of cathepsin B (CTSB) and cathepsin D (CTSD) were based on the housekeeping control α-tubulin; the densitometry results (**c**) were normalized to the quantity of α-tubulin and compared by unpaired two-tailed Student’s t test. *n* = 5 for each group. **d**, **e** Representative confocal immunofluorescence images showing the tissue distribution of two cathepsin members (red), i.e., CTSB (d) and CTSD (**e**) in liver sections of *Prep*^+/-^ and *Prep*^-/-^ mice treated with WD/CCl_4_ or ND/Oil. Macrophages were labeled by IBA1 immunoreactivity (green). Nuclei were labeled by DAPI (blue). Scale bar indicates 50 μm. Data information: Bars represent the mean ± SEM. *p < 0.05, **p < 0.01.

Cathepsin B (CTSB) and cathepsin D (CTSD) are the two most abundant cathepsins in lysosomes and have been reported to play profibrogenic roles in liver fibrosis^37,38^; therefore, we hypothesized that they might be the key to the mechanism underlying *Prep* knockout-induced exacerbated liver fibrosis. We first examined the hepatic distribution of CTSB and CTSD in the murine liver based on recently published scRNA-seq data^39^. Although cathepsins are widely expressed in nearly all liver cells that possess lysosomes^37^ and their expression in hepatic stellate cells (HSCs) is elevated during HSC activation, according to a previous study^40^, we found that cathepsin B and D were predominantly expressed in macrophages in healthy and NAFLD mouse livers (Supplementary Fig. 5a-b). Therefore, macrophages could be regarded as the main source of these two profibrogenic proteins during liver fibrosis, which in turn indicates that the expression levels of CTSB and CTSD in homogenized liver tissue samples may roughly reflect their levels in hepatic macrophages in vivo. Therefore, we measured the mRNA levels of CTSB and CTSD and found them to be upregulated markedly in WD/CCl_4_-treated mice of both genotypes, and more importantly, their levels were further increased by *Prep* knockout (Fig. 7a). The higher levels of CTSB and CTSD in *Prep*^-/-^ mice compared with *Prep*^+/-^ mice were further validated at the protein level (Fig. 7b, c).

We further examined the histological distribution of CTSB and CTSD in mouse livers. We found that CTSB and CTSD in healthy mouse livers were mainly distributed in the cytoplasm of macrophages, where they appeared as large spots, and were also distributed in nonmacrophage cells, where they appeared as small spots with low fluorescence intensity (Fig. 7d, e). However, in WD/CCl_4_-induced fibrotic livers, in addition to the small spots with low fluorescence intensity, we found large ring-like aggregates of CTSB and CTSD with high immunofluorescence signals deposited in fibrotic lesions; these ring-like aggregates was larger in *Prep*^-/-^ mice than in *Prep*^+/-^ mice (Fig. 7d, e). We zoomed in and found that these aggregates were predominantly colocalized with or were in close proximity to macrophages (Supplementary Fig. 5c), and did not colocalize with HSCs (Supplementary Fig. 5d-e), signifying that macrophages were the predominant sources of these aggregates in liver fibrosis.

**Fig. 8:**
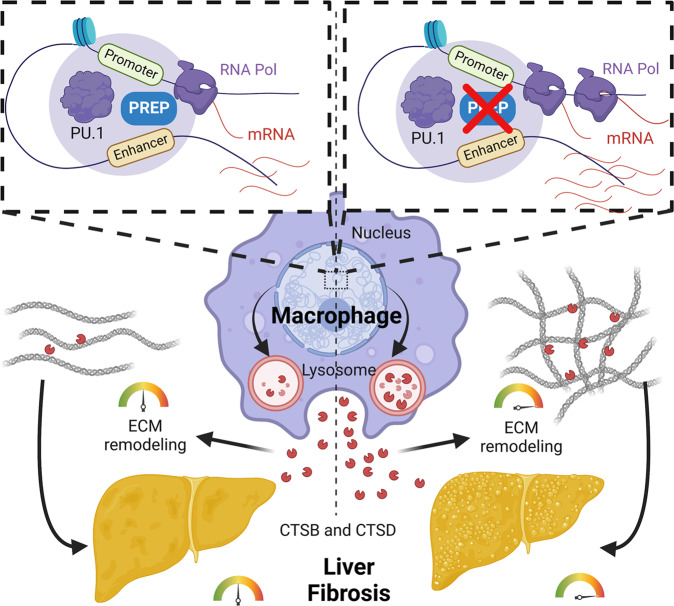
Schematic showing PREP-mediated transcriptional regulation in hepatic macrophages during liver fibrosis (created with BioRender.com). PREP prolyl endopeptidase, Pol polymerase, ECM extracellular matrix, CTSB cathepsin B, and CTSD cathepsin D.

Taken together, our findings indicate that PREP in macrophages serves as a transcriptional coregulator that interacts with the transcription factor PU.1 and regulates active *cis*-regulatory genomic regions. The transcriptional regulatory activity of PREP inhibits profibrogenic cathepsin B and D expression, thus constraining pathological fibrosis progression (Fig. 8).

## Discussion

Transcriptional regulation in macrophages largely determines the state and function of macrophages^7–9^, but related molecular mechanisms have not been fully elucidated. Recent studies have increased the appreciation for transcription-related dogmas and highlight the role of transcriptional coregulators in fine-tuning of transcription^41,42^. In this study, we found that PREP in macrophages could remodel the cellular transcriptome and functions, partially inhibiting the development of fibrosis. We identified that nucleus-localized PREP in macrophages functions as a transcriptional coregulator: it is mainly distributed in active *cis*-regulatory genomic regions, and physically interacts with the transcription factor PU.1. Our data revealed that PREP plays a crucial role in regulating macrophage functions and provides new insights into transcriptional mechanisms and fibrosis-related pathophysiology.

Transcriptional coregulators are proteins that exhibit transcription regulation-related activity without binding to DNA per se; in contrast, they bind with DNA-bound transcription factors to mediate downstream gene regulation^42^. Well-characterized transcriptional coregulators include steroid receptor coactivator (SRC) family members, which are coregulators of nuclear receptors^41,43^. However, transcriptional coregulators with crucial transcription-related functions for other transcription factors remain to be discovered. In this study, a novel role for PREP as a transcriptional coregulator in remodeling the macrophage transcriptome is reported for the first time. We proposed it based on the following evidence: (1) PREP in macrophages is predominantly localized to the nucleus and mainly distributed in TSS-adjacent *cis*-regulatory regions (Fig. 3); (2) PREP in macrophages physically interacts with the transcription factor PU.1, and is distributed in genomic regions enriched with PU.1 motif sequences and bound by PU.1 (Fig. 4); and (3) PREP is absent for currently appreciated DNA-binding-related structure^20^. These characteristics coincide with the current theory of transcriptional coregulators, i.e., “*do not bind DNA directly but are recruited by TFs to specific genomic regulatory loci*”^42^. Therefore, the discovery of PREP as a novel transcriptional coregulator would help to expand current understanding of the transcriptional network. Considering the marked change in the transcriptome induced by *Prep* ablation (Fig. 1) and the high number of PREP direct downstream genes (Fig. 5), the influence of PREP-mediated transcriptional regulation should not be neglected, at least not in macrophages.

PREP was traditionally recognized as a cytosolic member of the DPP family with hydrolytic activity in processing immunoactive peptides and neuropeptides^19^. However, nonproteolytic functions of PREP mediated through its physical interaction with other proteins (e.g., α-synuclein) have been discovered in recent studies^20^. In addition, the nuclear localization of PREP in cells including fibroblasts, germ cells and cerebellar granule cells has been sporadically reported^36,44,45^, but the corresponding biological functions related to this nuclear localization has remained elusive and unexplored. In this study, we identified a novel function of PREP as a transcriptional coregulator that interacts with the transcription factor PU.1 in transcriptional regulation-related nuclear condensates (Figs. 3 and 4). In fact, PREP consists of two main domains, a hydrolase catalytic domain and a seven-bladed β-propeller domain^20^. Although the β-propeller domain in PREP has been proposed to be a substrate-gating filter for the hydrolase catalytic domain^46^, the β-propeller domain is a conserved structure in numerous proteins with versatile functions, including providing a platform for protein-protein interactions^47^. Therefore, PREP may interact with other proteins, including transcription factors, through its β-propeller domain to regulate transcription. In eukaryotic cells, many proteins have been found to exhibit more than one unique biological activity, a phenomenon called “protein moonlighting”^48,49^. In the era of transcriptional regulation, for instance, some histone-modifying proteins have been found to possess noncatalytic functions independent of their enzymatic activities^50^. Our findings indicate that PREP may also be a multifunctional protein that plays dual roles in peptide processing and transcriptional regulation, which broadens the current understanding of PREP per se and transcriptional regulatory proteins.

Although immune activation of macrophages relies on extracellular signal-activated signal-transduction to be initiated, the ultimate intensity of immune responses needs to be fine-tuned by transcriptional regulatory network and epigenetic modifications^51^. Numerous functional gene modules in macrophages also need to be differentially regulated in accordance with the microenvironment^2,52^. In this study, *Prep* ablation contributed to significant changes in the transcriptome, especially the expression of M1/M2-related markers, during M1/M2 polarization (Fig. 1). Interestingly, the effect of *Prep* knockout on M1/M2 polarization marker expression varied among different specific markers, as exemplified by upregulated *Nos2* in contrast with downregulated inflammatory triads under M1 polarization, as well as upregulated *Arg1* and *Mrc1* in contrast with downregulated *Chil3* and *Retnla* (Fig. 1). These seemingly contradictory results indicate that PREP-mediated transcriptional regulation ultimately leads to the differential regulation of different functional modules under the same macrophage activation, which coincides with *Prep* knockout-induced alterations in multiple immune-related functional gene modules (Fig. 5). More importantly, we found that *Prep* ablation induced a marked increase in phagocytosis and invasion ability of M2-polarized macrophages (Fig. 6), indicating that PREP-mediated transcriptional regulation in functional gene modules were sufficient to induce profound alterations in corresponding functional phenotypes of macrophages. One of the physiological functions of transcriptional coregulators is to preferentially modulates genes in several specific gene modules among all the downstream modules of a transcription factor^42^. Based on this notion, PREP may serve as a transcriptional coregulator with a preferential regulatory effect on some PU.1 downstream modules, and is therefore a promising target for fine-tuning of macrophage immune activation.

Fibrosis is a key pathophysiological process that involves the substantial participation of aberrantly activated macrophages in close proximity to fibrotic regions in tissues^53–55^. The molecular and functional features of these fibrosis-related macrophages can be partially reproduced during M2 polarization in vitro^34^. ECM remodeling-related proteases in macrophages not only facilitate cell migration and invasion through the ECM, but also are profibrogenic factors by remodeling the excessively produced ECM protein into disorganized ECM in fibrotic tissue^10,56,57^. In this study, we found that genes encoding lysosomal proteins, as well as cellular functions including phagocytosis and cell invasion, were collectively upregulated after *Prep* knockout in M2-polarized macrophages (Fig. 6), indicating a repressive role for PREP in lysosome-related functional modules, including ECM remodeling. Lysosomes are highly enriched with enzymes. These enzymes can facilitate the degradation of macromolecules via phagocytosis, as well as cell invasion and ECM remodeling after being secreted into the extracellular space^35,58^. Among these lysosomal genes, genes encoding two established profibrogenic proteases, cathepsin B and D^38^, were overexpressed in *Prep*^-/-^ M2-polarized BMDMs and *Prep* knockdown NIH-3T3 cells, and cathepsin B and D were morbidly aggregated in fibrotic regions of WD/CCl_4_-induced fibrotic murine liver (Figs. 6 and 7). Correspondingly, liver fibrosis was exacerbated in WD/CCl_4_-induced fibrotic murine livers (Fig. 2). These results indicate that PREP is crucial for inhibiting cathepsin-mediated pathological ECM remodeling in multiple cell types, especially macrophages, which are the main source of fibrosis-related cathepsins. Although PREP-mediated transcriptional regulation seems to exert a permissive effect on inflammation, as signified by macrophage proinflammatory M1 polarization (Figs. 1 and 5) and our previous studies in NASH liver inflammation murine models^25,26^, the key pathological process underlying fibrosis progression is aberrant tissue repair (e.g., fibrogenesis and ECM remodeling) during intermissions of repetitive inflammation, but not inflammation episodes per se^59^. Considering the preferential regulatory effect of PREP on different functional modules, we propose that PREP in macrophages in a pathological microenvironment serves as a transcriptional coregulator that not only supports proinflammatory responses during inflammation but also hinders ECM remodeling during fibrosis. Additionally, based on clinical evidence, circulatory PREP activity has been found to be strongly and negatively correlated with cirrhosis prognostic scores in cirrhotic patients^60^. Collectively, these evidences indicate that the pathogenesis of fibrosis might involve insufficient PREP-mediated transcriptional repression on cathepsin B and D expression in macrophages.

The major limitation of this study is the limited appreciation of the underlying biochemical functions of PREP behind its role as a transcriptional coregulator, as this is beyond our current technical ability. In addition, global knockout of PREP is not as powerful as macrophage-specific knockout in validating the predominant role of macrophages in PREP/cathepsin axis-related aggravated liver fibrosis. Moreover, the liver fibrosis mimicked by the WD/CCl_4_ model did not fully recapitulate fibrosis in NASH because of differences in the tissue injury for eliciting fibrotic processes.

In summary, the data in this study have revealed a novel molecular function for PREP as a transcriptional coregulator of the transcription factor PU.1 in macrophages. PREP-mediated transcriptional regulation markedly remodels the transcriptome of macrophages and alters their functions, including suppressing the expression of the profibrogenic proteins cathepsin B and D and consequently inhibiting fibrosis. The discovery of PREP as a transcriptional coregulator in macrophages indicates that it is a promising molecular target for fine-tuning macrophage activation and deepens the current understanding of NASH-related fibrosis pathophysiology.

## Supplementary information

Supplementary file

## Data Availability

The genomics data were deposited in the NCBI Gene Expression Omnibus (https://www.ncbi.nlm.nih.gov/geo/) and are accessible through the GEO Series accession number GSE213051. All other data supporting the findings of this study are available from the corresponding authors on reasonable request.
