# Supplementary material for: Prolyl endopeptidase remodels macrophage function as a novel transcriptional coregulator and inhibits fibrosis
Source: Exp Mol Med. 2023 Jul 3;55(7):1437–50. doi: 10.1038/s12276-023-01027-8 (PMC10394032; doi:10.1038/s12276-023-01027-8)
Supplement: Supplementary file 1 — Supplementary file [file 12276_2023_1027_MOESM1_ESM.pdf]

Prolyl endopeptidase remodels macrophage function as a novel transcriptional coregulator and inhibits fibrosis

Shuang-Zhe Lin[a,†], Wei-Jie Wu[a,b,†], Yu-Qing Cheng[a], Jian-Bin Zhang[a], Dai-Xi Jiang[a,c], Tian-Yi Ren[a,d], Wen-Jin Ding[a], Mingxi Liu[e*], Yuan-Wen Chen[f,g*] and Jian-Gao Fan[a,d*]

This PDF file includes:

Supplementary Materials and Methods

Supplementary Figures

Supplementary Materials and Methods

RNA-seq library preparation and sequencing

Total RNA was isolated via TRIzol reagent according to the manufacturer's protocol and used for subsequent RNA-seq analysis. 500 ng RNA per sample was used as input material for the RNA sample preparations. Sequencing libraries were subsequently constructed using TruSeq Stranded mRNA LTSample Prep Kit (Illumina, San Diego, CA, USA) according to the manufacturer's instructions. Paired-end sequencing was carried out with the Illumina NovaSeq 6000 with read length of 150 bp. About 50 million raw reads for each sample were generated.

ChIP-seq data analysis pipeline

H3K27ac and PU.1 ChIP-seq data of differently polarized BMDMs were generated by a previous research[1] (GSE38377). Single-end reads ChIP-seq were aligned to mm10 genome using bwa (v0.7.17.4). Deeptools (3.3.2) was used to generate the read coverage track (command bamCoverage with normalization by RPKM). Peak calling for H3K27ac and PU.1 was performed to generate bedGraph files using macs2(v2.1.4). The bedGraph files containing signal per million reads were then compared to derive differential ChIP-seq peaks by using command bdgdiff from macs2. Command plotProfile and plotHeatmap in deeptools were used to visualize the H3K27ac and PU.1 distribution over regions centered by H3K27ac and PU.1 peaks respectively.

scRNA-seq data analysis pipeline

scRNA-seq data of liver from mice on either standard diet or western diet were generated by a previous research[2] (GSE192742) and are available for additional analysis and download at www.livercellatlas.org. The preprocessed data with annotated cell-identity information generated by the original research were downloaded at www.livercellatlas.org. DotPlot made by package Seurat (4.3.0) was used to visualize the gene expression pattern in different cell clusters.

Serum biochemical analysis

The serum levels of alanine aminotransferase (ALT) and aspartate aminotransferase (AST) were determined by ALT activity assay kit (MAK052, Sigma Aldrich) and AST activity assay kit (MAK055, Sigma Aldrich), respectively.

RT-qPCR

Total RNA was isolated via TRIzol reagent according to the manufacturer's protocol. RNA was reverse transcribed to cDNA using the PrimeScript™ RT Master Mix kit (TaKaRa, RR036A). The cDNA was diluted 10 times and used as RT-qPCR template. cDNA was amplified and quantified in QuantStudio3 Real-Time PCR system using the qPCR SYBR® Green Master Mix (Yeasen, 11199ES08). The relative expression levels of *Col1a1*, *Col1a2*, *Ctsb*, *Ctsd*, *Ctsk* and *Ctss* were calculated relative to *Gapdh* using the comparative cycle threshold method. The primer sets are as follows: *Col1a1*, 5′-GCTCCTCTTAGGGGCCACT-3′ and 5′-CCACGTCTCACCATTGGGG-3′; *Col1a2*, 5′-GTAACTTCGTGCCTAGCAACA-3′ and 5′-CCTTTGTCAGAATACTGAGCAGC-3′; *Ctsb*, 5′-TCCTTGATCCTTCTTTCTTGCC-3′ and 5′-ACAGTGCCACACAGCTTCTTC-3′; *Ctsd*, 5′-CGATTATCAGAATCCCTCTGCG-3′ and 5′-GGTCTTAGGCGATGACTGCAT-3′; *Ctsk*, 5′-GAAGAAGACTCACCAGAAGCAG-3′ and 5′-TCCAGGTTATGGGCAGAGATT-3′; *Ctss*, 5′-CCATTGGGATCTCTGGAAGAAAA-3′ and 5′-TCATGCCCACTTGGTAGGTAT-3′; primer set for GAPDH (CAT# B661304-0001) was purchased from Sangon Biotech (Shanghai, China).

Immunoblotting and immunoprecipitation

The liver tissue samples were homogenized in RIPA buffer containing 1% PMSF. The protein samples were separated on 10% SDS-PAGE gel, and electroblotted onto PVDF membranes. The membranes were blocked for 1 h at RT in 5% w/v non-fat dry milk in TBST buffer, and incubated overnight at 4°C with the following primary antibodies: PREP (Abcam, ab58988), 1:1000; α-SMA (Abcam, ab7817), 1:1000; Cathepsin B (Abcam, ab214428), 1:2000; Cathepsin D (Abcam, ab75852), 1:2500; and α-Tubulin (1E4C11; ProteinTech, 66031-1-Ig), 1:2500. After washing in TBST, the blots were incubated with HRP-conjugated anti-rabbit or mouse IgG. The signal was detected with the Immobilon Western HRP Substrate (Millipore, WBKLS0100) using the Amersham Imager 680 (GE Healthcare).

For PREP immunoprecipitation, cells were lysed in cold RIPA buffer supplemented with Protease inhibitor cocktail (Beyotime, P1010), sonicated and centrifuged. Supernatants were then incubated with primary antibody of PREP (Abcam, ab58988) for 1 hour at RT. Protein A magnetic beads (Beyotime, P2102) were added and rotated

at RT for 1 hour. The beads were then washed 3 times with TBST, resuspended in reducing SDS gel loading buffer, and heated at 95ºC for 10 minutes. The immunoprecipitated proteins were subsequently analyzed by western blotting with the primary antibodies for PREP (Abcam, ab58988) and PU.1 (Abcam, ab227835).

Histological analysis

For general histological pathology and fibrosis detection, formalin-fixed liver tissue samples were embedded in paraffin and 4 μm sections and stained with Hematoxylin and Eosin (HE) and Sirius Red according to standardized protocols. Images were captured by using Olympus BX51 microscope. Quantification of Sirius red positive staining of liver sections was performed by using ImageJ (v1.53c).

For histological immunofluorescence, paraffin-embedded mouse liver tissue sections were deparaffinized and rehydrated, followed by antigen retrieval in citrate solution for 15 min. Then the sections were covered with 5% BSA in PBS at room temperature (RT) for 30 min before antibody incubation.

For dual labelling of cathepsin and macrophage, the sections were co-incubated with mouse anti-IBA1 (1:100, Abcam, ab283319) and rabbit anti-CTSB (1:200, Abcam, ab214428) or anti-CTSD (1:200, Abcam, ab75852) at 4 °C overnight followed by incubation with CoraLite488-conjugated donkey anti-mouse IgG (1:100, ProteinTech, SA00013-5) and Cy3-conjugated donkey anti-rabbit IgG (1:100, Servicebio, GB21403) at room temperature for 1 h. The sections were mounted with antifade mounting medium with DAPI (Beyotime) for nuclear staining.

For triple labelling of cathepsin, macrophage and hepatic stellate cells (HSCs), the sections were firstly incubated with rabbit anti-Desmin (1:200, Abcam, ab15200) at 4 °C overnight followed by incubation with HRP-conjugated anti-rabbit IgG. Then FITC-Tyramide (Servicebio, G1222) was used according to the manufacturer's instructions for fluorescent labeling of desmin. Second-round antigen retrieval in citrate solution was implemented to remove anti-Desmin and anti-rabbit antibodies, followed by co-incubated with mouse anti-IBA1 (1:100, Abcam, ab283319) and rabbit anti-CTSB (1:200, Abcam, ab214428) or anti-CTSD (1:200, Abcam, ab75852) at 4 °C overnight, as well as incubation with Dylight405-conjugated donkey anti-rabbit IgG (1:100, Yeasen,

34204ES60) and Cy3-conjugated goat anti-mouse IgG (1:200, Servicebio, GB21301) at room temperature for 1 h.

The fluorescence images were captured by using a Leica TCS SP8 confocal microscope system.

Immunocytochemistry

BMDMs and RAW 264.7 cells were fixed with 4% paraformaldehyde, permeabilized with immunostaining permeabilization buffer (Beyotime) and blocked with 5% bovine serum albumin (BSA) in PBS. Afterwards, cells were sequentially incubated with anti-PREP (1:200, Abcam, ab58988) at 4°C overnight and Alexa Fluor 488 goat anti-rabbit (1:100, Abcam, Cat# ab150077) or Alexa Fluor 594 goat anti-rabbit (1:100, CST, 8889) IgG secondary antibody for 1 h at room temperature. The coverslips were mounted with antifade mounting medium with DAPI (Beyotime) for nuclear staining. For mitochondrial labelling, cells were cultured in the culture medium with 200 nM Mitotracker Red CMXRos (Invitrogen) for 15 min prior to fixation of cells. Images were captured by using a Leica TCS SP8 confocal microscope system.

Small interfering RNA (siRNA) treatment

The siRNA targeting PREP (sense: CCCGUAUUCUUGGCUUGAATT, anti-sense: UUCAAGCCAAGAAUACGGGTT) and the negative control (sense: UUCUCCGAACGUGUCACGUTT, anti-sense: ACGUGACACGUUCGGAGAATT) at the concentration of 20 μM were obtained from GenePharma. Transfection was performed using Advanced DNA/RNA Transfection Reagent (Zeta Life) according to the manufacturer's instructions. Briefly, NIH-3T3 cells in 12-well plates were transfected with siRNA targeting *Prep* negative control (5 μl/well) for 24 h.

Plasmid constructs and luciferase assay

For construction of *Ctsb* promoter reporter vector, a dual-luciferase reporter vector was first constructed by cloning *hRluc*-neo gene and upstream promoter from pmirGLO vector into pGL3-Basic vector (Promega). The promoter region of murine *Ctsb* (−262 to 538 bp) was then synthesized by Sangon Biotech and cloned into the reporter vector. For construction of PREP overexpression plasmid, cDNA of murine PREP was cloned into pcDNA3.1-MCS-EF1-ZsGreen1 (Genomeditech). Transfection was performed using Advanced DNA/RNA Transfection Reagent (Zeta Life) according to the

manufacturer's instructions. For gene expression detection, NIH-3T3 cells in 12-well plates were transfected with PREP overexpression or control plasmid (4 μg/well) for 24 h. For luciferase assay, NIH-3T3 cells in 24-well plates were transfected with PREP overexpression or control plasmid (2 μg/well) for 24 h, followed by transfection with *Ctsb* promoter reporter vector (2 μg/well) for 24 h; then cell extracts were prepared, and the luciferase activity was measured by the Dual Luciferase Reporter Gene Assay Kit (Yeasen, 11402ES60).

Invasion assay

The cell invasion assay was performed using a 24-well Transwell chamber with 8 μm pore size insert and pre-coated Matrigel Matrix (356234, Corning). BMDMs were harvested and suspended in 200 μl serum-free RPMI 1640, then 5×10^4 cells were seeded into upper chamber. The lower chamber was filled wsith 600 μl RPMI 1640 containing 10% FBS. After incubation in 5% CO2 incubator for 24 hours, the non-invading cells on upper surface were removed by wiping the membrane with a cotton-tipped swab. The invading cells on the lower surface were fixed with 4% paraformaldehyde and stained with a 0.5% crystal violet solution. Images were randomly captured by using an Olympus IX73 inverted microscope system. The number of cells was counted in three randomly selected fields.

Phagocytosis assay

Phagocytosis was assessed with the Phagocytosis Assay Kit (Cayman Chemical, 500290). BMDMs were incubated for 12 h at 37 °C with FITC-labeled rabbit-IgG latex beads (1:200). Then the cells were harvested, suspended in PBS and measured with the Cytoflex S flow cytometer (Beckman). By using FlowJo 10, the singlets were gated, and median fluorescence intensity (MFI) on FITC channel was quantified. The relative MFI was calculated as the ratio between the MFI of each latex-bead-treated cell sample against the MFI of the cell sample without latex bead treatment.

Reference

1. Ostuni, R. *et al.* Latent enhancers activated by stimulation in differentiated cells. *Cell* **152**, 157-71 (2013)
2. Guilliams, M. *et al.* Spatial proteogenomics reveals distinct and evolutionarily conserved hepatic macrophage niches. *Cell* **185**, 379-96.e38 (2022)

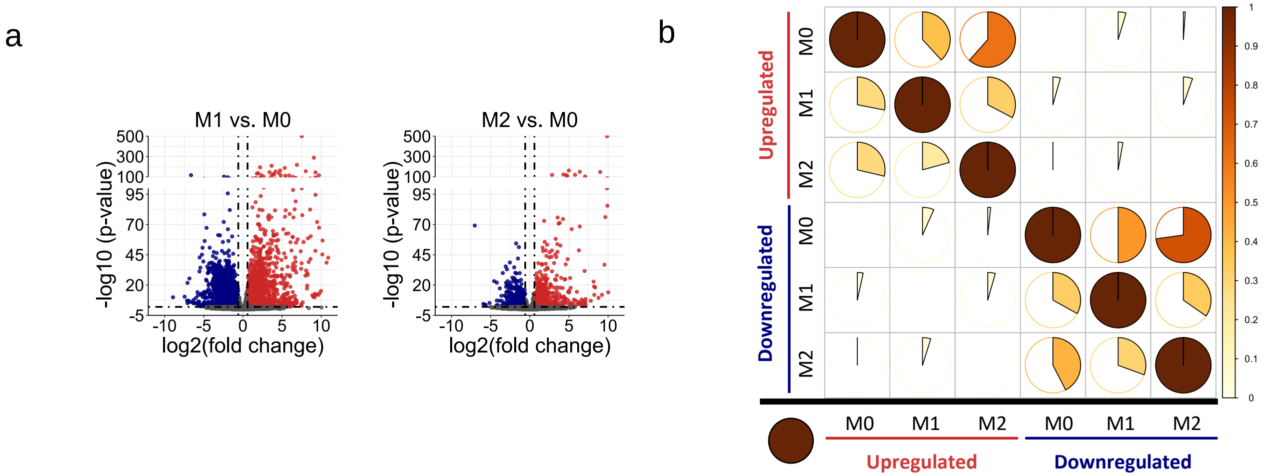

Supplementary Fig. 1 Changes in the transcriptome of murine bone marrow-derived macrophages (BMDMs) under M1/M2-polarization and *Prep* gene ablation.

(a) Volcano plots for differentially expressed genes (DEGs) between M1 and M0 Prep$^{+/-}$ BMDMs (left panel), as well as between M2 and M0 Prep$^{+/-}$ BMDMs (right panel).

(b) Pie plots showing the proportion of shared genes between two DEG sets (Prep-knockout-induced upregulated or downregulated DEGs under M0, M1 or M2 state) in either gene sets.

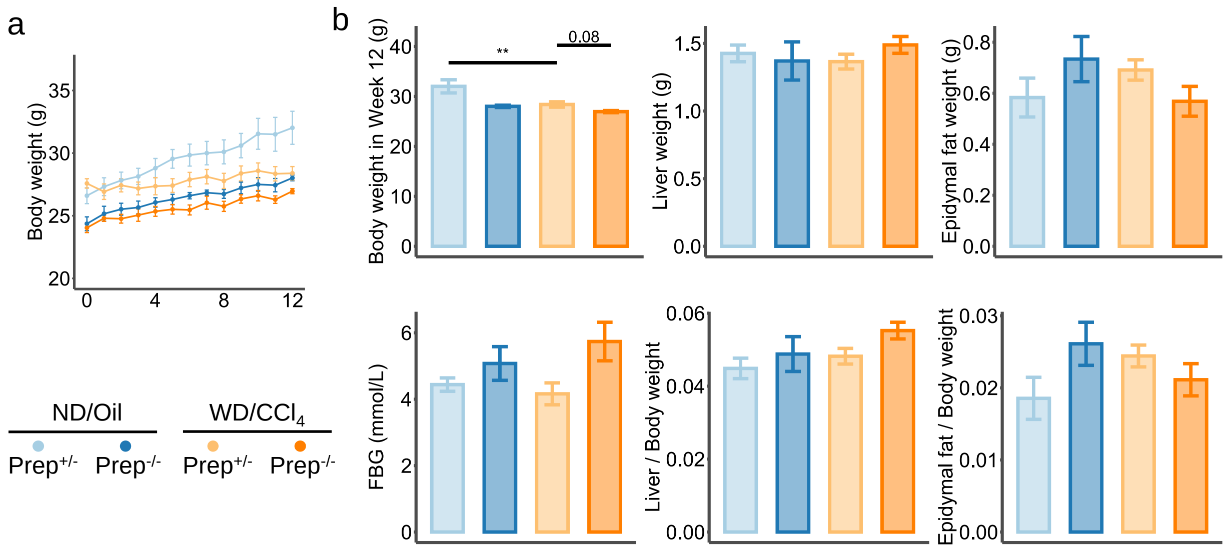

Supplementary Fig. 2 Changes in metabolic indices of mice with WD/CCl_4 treatment and/or *Prep* gene ablation.

(a) Changes in body weight of Prep$^{+/-}$ and Prep$^{-/-}$ mice treated with WD/CCl_4 or ND/Oil for 12 weeks.

(b) Body weight in week 12, liver weight, epidymal fat weight, fasting blood glucose (FBG), liver per body weight ratio and epidymal fat per body weight ratio in Prep$^{+/-}$ and Prep$^{-/-}$ mice treated with WD/CCl_4 or ND/Oil. Results were compared by one-way ANOVA and LSD (least significance difference) post-hoc test. Data information: n = 10 for WD/CCl_4-treated Prep$^{+/-}$ mice group, n = 8 for WD/CCl_4-treated Prep$^{-/-}$ mice group, n =5 for ND/Oil-treated groups. Bars represent mean ± SEM. *$p < 0.05$, **$p < 0.01$.

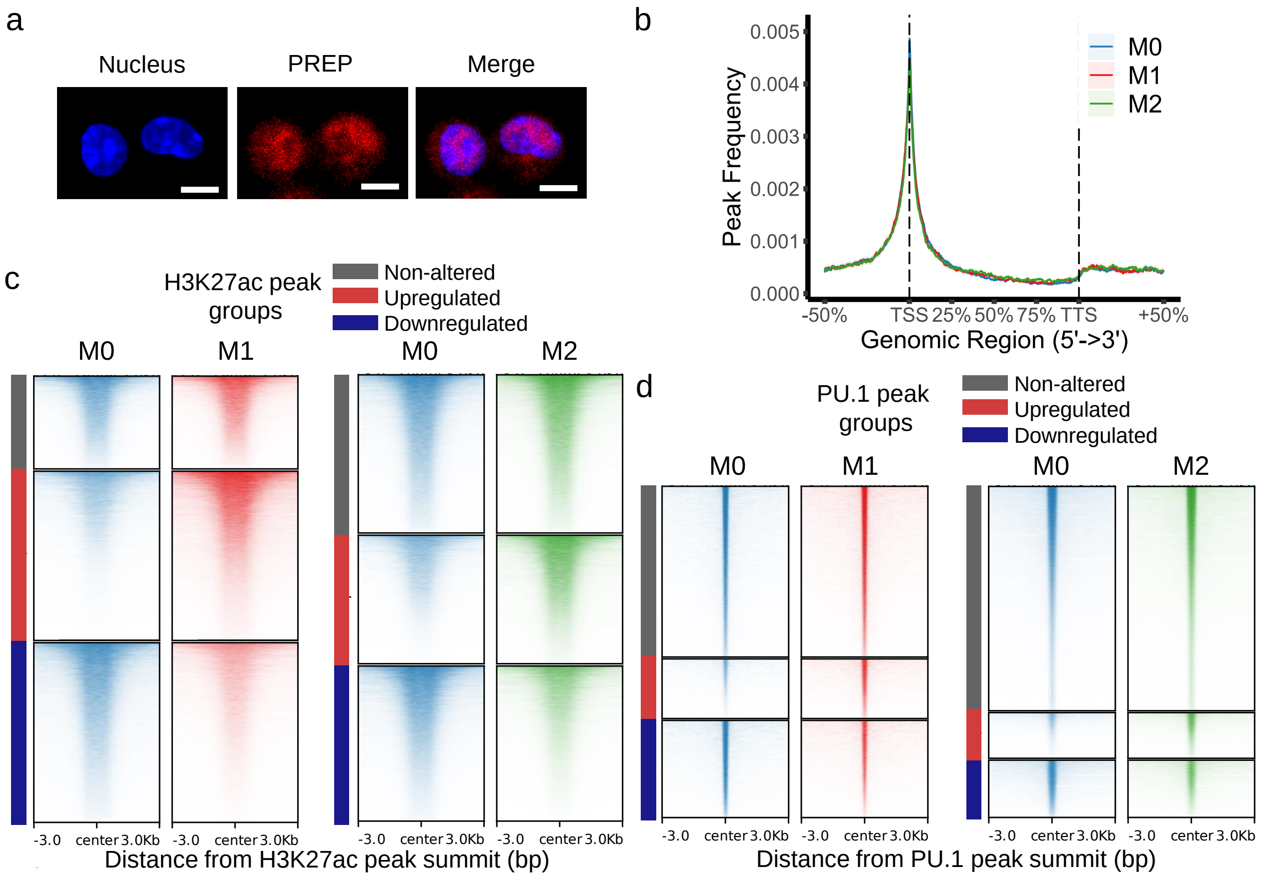

Supplementary Fig. 3 The nuclear localization of prolyl endopeptidase is associated with transcription regulation-related genomic regions in macrophages.

(a) Confocal immunofluorescence images showing the subcellular localization of prolyl endopeptidase (PREP) in unstimulated RAW264.7 cells. Nucleus are labeled by DAPI (blue). Scale bar indicates 5 μm.

(b) Metagene profile of PREP CUT&Tag peaks around genic regions in BMDMs under M0, M1 or M2 state. TSS, transcription start site; TTS, transcription termination site.

(c-d) The average enrichment profile and heatmap of H3K27ac (c) and PU.1 (d) ChIP ± 3kb around differential H3K27ac ChIP peaks during M1/M2 polarization of BMDMs.

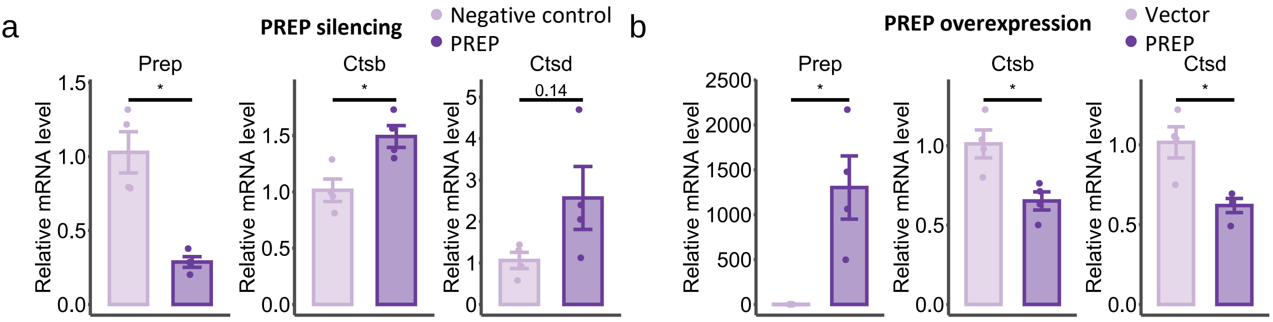

Supplementary Fig. 4 PREP inhibits *Ctsb* and *Ctsd* expression in NIH-3T3 cells.

(a-b) Relative mRNA levels of *Prep*, *Ctsb* and *Ctsd* in NIH-3T3 cells transfected with siRNA targeting PREP or negative control siRNA (a), and in NIH-3T3 cells transfected with PREP overexpression plasmid or control plasmid (b). Results were compared by unpaired two-tailed Student's t-test.

Data information: n = 4 for each group. Bars represent mean ± SEM. *p < 0.05.

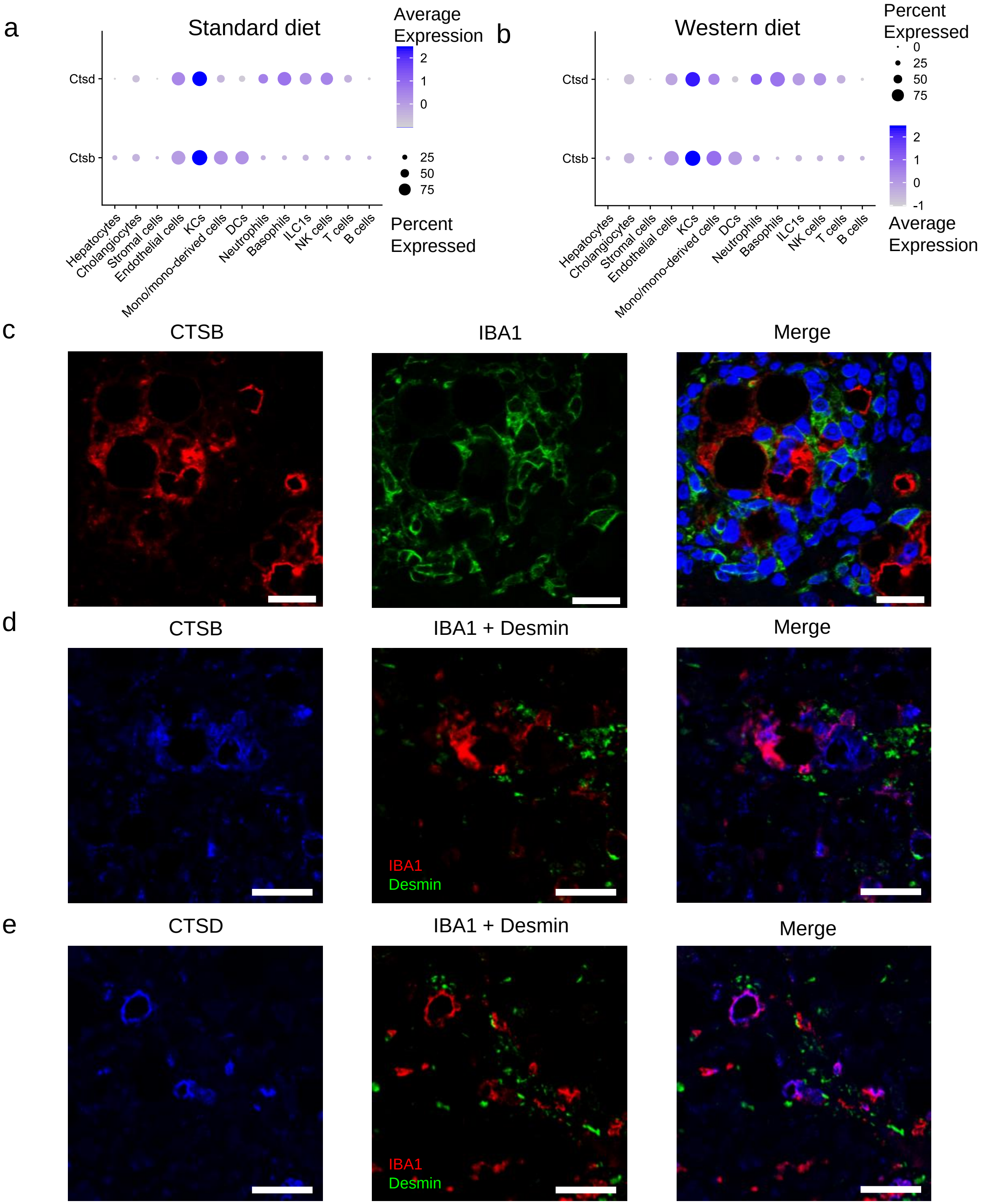

Supplementary Fig. 5 Cathepsin B and cathepsin D in liver microenvironment are predominantly expressed and secreted by macrophages.

(a-b) Ctsb and Ctsd expression in scRNA-seq of liver from mice on either standard diet or western diet.

(c) Zoom-in representative immunofluorescence image showing the close proximity of the aggregated cathepsin B (red) and IBA1-labeled macrophages (green) in liver section of Prep$^{-/-}$ mice treated with WD/CCl_4. Nucleus are labeled by DAPI (blue). Scale bar indicates 15 μm.

(d-e) Representative immunofluorescence image showing the tissue distribution of two cathepsin members (blue), i.e. CTSB (d) and CTSD (e) in liver sections of mice treated with WD/CCl_4. Macrophages and hepatic stellate cells are labelled by IBA1 (red) and Desmin (green). Scale bar indicates 30 μm.
